# Nicotine and the cardiovascular system: unmasking a global public health threat

**DOI:** 10.1093/eurheartj/ehaf1010

**Published:** 2025-12-18

**Authors:** Thomas Münzel, Filippo Crea, Sanjay Rajagopalan, Thomas Lüscher

**Affiliations:** Department of Cardiology, University Medical Center Mainz Germany, Johannes Gutenberg University, Langenbeckstrasse 1, Mainz 55131, Germany; Center of Excellence of Cardiovascular Sciences, Ospedale Isola Tiberina, Gemelli Isola, Rome, Italy; Department of Cardiovascular and Pulmonological Sciences, Catholic University of the Sacred Heart, Rome, Italy; University Hospitals, Harrington Heart and Vascular Institute, 11100 Euclic Ave, Cleveland, OH, USA; Case Western Reserve University School of Medicine, 10900 Euclid Ave, Cleveland, OH, USA; Heart Division, Royal Brompton & Harefield Hospitals, London, UK; King’s College, Cardiovascular Academic Group and Imperial College, National Heart and Lung Institute, London, UK; Center for Molecular Cardiology, University of Zurich, Wagistrasse 12, 8952 Schlieren, Switzerland

**Keywords:** Cardiovascular prevention, Smoking, Nicotine, E-cigarettes, Oxidative stress, Inflammation, Endothelial dysfunction, Policy messages

## Abstract

Nicotine-containing products, whether combustible or smokeless, pose a growing threat to cardiovascular (CV) health. While tobacco smoking continues to cause millions of deaths annually, the rapid uptake of e-cigarettes, heated tobacco, and synthetic nicotine pouches, particularly among the youth, risks reversing decades of progress in tobacco control. In this policy statement, 12 evidence-based key messages that highlight the CV toxicity of nicotine are presented, irrespective of delivery system, and the urgent need for comprehensive regulations. These messages address the vascular and metabolic harms of nicotine, the dangers of passive exposure, the key pathophysiological pathways leading to CV morbidity and mortality, and the economic burden of nicotine-induced CV disease. Importantly, this is the first expert consensus paper to address nicotine itself as a direct CV toxin, independent of combustion. Special attention is given to the paediatric addiction crisis, driven by flavoured products and aggressive marketing, and to the misleading narrative of ‘safer nicotine.’

This paper appears at a critical regulatory turning point: the *European Commission’s revised Tobacco Taxation Directive* (July 2025), which for the first time introduces minimum excise duties on e-liquids, heated tobacco, and nicotine pouches. While this directive represents an important step, it must be complemented by broader regulations, including advertising bans, flavour restrictions, and indoor-use prohibitions, applied consistently across all nicotine products.

Together, the 12 key messages provide a policy blueprint to protect CV health and prevent the rise of a new generation of nicotine-addicted youth.

## Graphical Abstract

**Graphical Abstract:**
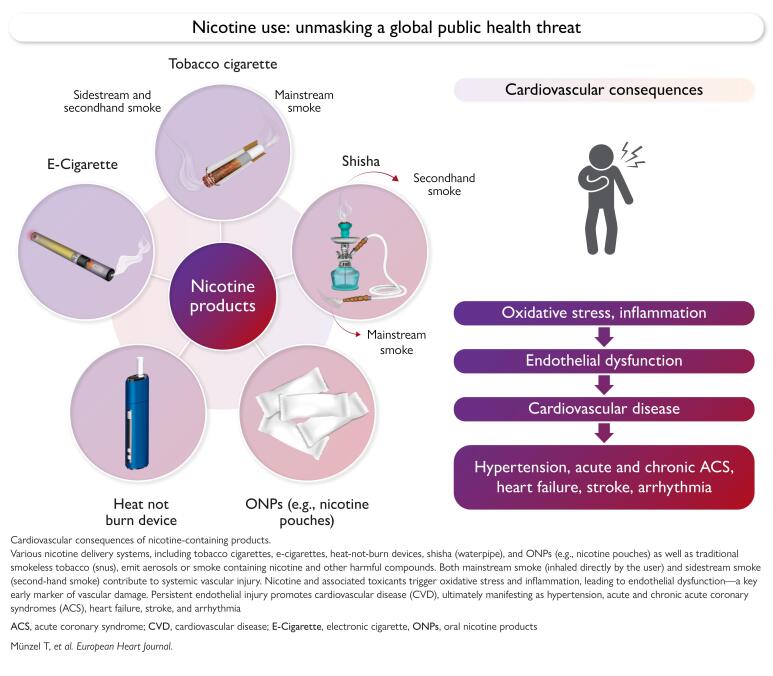

## Introduction

This policy paper presents 12 key messages that provide a clear, evidence-based framework for tackling the cardiovascular (CV) harms of nicotine products. These messages are designed to guide policymakers through the complex and rapidly evolving landscape of nicotine regulation, covering both combustible and non-combustible products (*Table 1*). This is the first Expert Consensus paper explicitly addressing nicotine itself as a CV toxin, providing novelty and urgency to our recommendations. Each message is discussed in detail, highlighting the scientific rationale and the regulatory implications.

**Table 1:** Twelve policy-driven key messages, each grounded in evidence and designed to resonate with legislators, ministries of health, and public health agencies

No.	Title	Policy message summary
Part I: What are the consequences of nicotine consumption		
1	Nicotine-Related CV Disease as a Leading Global Health Burden	Nicotine-related products, including e-cigarettes and oral pouches, cause mil-lions of deaths annually, mostly due to CV disease, and will endanger public health without regulation.
2	Nicotine is a Powerful CV Toxin—Regardless of Delivery Method	Nicotine activates the sympathetic nervous system, increases blood pressure, causes endothelial dysfunction, and accelerates atherosclerosis, even in non-combusted forms.
3	No Nicotine-Containing Product Is Safe for the Heart or Blood Vessels	All forms of nicotine use, e.g. cigarettes, e-cigarettes, heat-not-burn devices, waterpipes, or oral pouches, impair vascular function and elevate CV risk.
4	E-Cigarettes Are Less Harmful Than Cigarettes—But Far from Harmless	E-cigarettes reduce exposure to some toxins, but still cause vascular damage, foster addiction, encourage dual use, and lack proven long-term safety.
5	Passive Nicotine Exposure: An Invisible CV Risk	Passive nicotine exposure be it cigarettes, e-cigarettes, waterpipes, and heated tobacco—causes immediate vascular harm and increases CV risk in non-users, especially children and vulnerable groups, demanding comprehensive smoke-free legislation.
6	The Vascular Red Flag: How Nicotine Disrupts Endothelial Health Across All Products	Nicotine, whether smoked, vaped, heated, or chewed, impairs endothelial function through oxidative stress and eNOS uncoupling, making endothelial dysfunction a powerful early warning sign and regulatory benchmark for cardiovascular harm.
7	The Hidden Bill: How Nicotine Harms Hearts—and Bleeds Public Budgets	Nicotine use not only causes persistent vascular damage and CV deaths but also imposes massive economic burdens on healthcare systems and national productivity, making strong nicotine regulation a fiscal as well as a public health imperative.
**Part II: Policy actions to address nicotine-induced adverse health effects**		
8	The Next Generation Hooked: How the Nicotine Industry Rebrands Addiction for Teens	Aggressively marketed flavoured nicotine products are fuelling a youth addiction crisis across Europe and North America, exploiting regulatory gaps and neuro-biological vulnerability, demanding urgent political action to prevent a new epidemic.
9	One Message, One Risk: No Safe Nicotine for the Cardiovascular System	Decades of accumulating evidence and global expert consensus confirm that all nicotine products, regardless of form or dose, pose serious CV risks, demanding unified, risk-based regulation that ends the illusion of ‘safer’ alternatives.
10	From Loopholes to Leadership: Turning Cardiovascular Science into Binding Nicotine Policy	Despite consistent scientific evidence, comprehensive policy measures, including closing regulatory gaps, harmonizing taxation across all nicotine products, and strengthening youth protection, are required to reduce the CV harms of nicotine and safeguard public health.
11	Fighting Passive Exposure: The Case for Total Smoke-Free Spaces	Comprehensive indoor and outdoor bans on smoking, vaping, and waterpipes are essential to protect public health, especially in urban and high-risk settings.
12	A Call to Action for Cardiologists and Policymakers	Cardiologists must integrate nicotine prevention into routine care, and policymakers must enact decisive, unified regulation to protect current and future generations from nicotine’s CV harms

This document comes at a pivotal moment: Across Europe, there is a renewed debate about increasing tobacco and e-cigarette prices, driven by growing concerns about uptake and long-term health impacts, particularly in the young. On 16 July 2025, the European Commission adopted a modernized Tobacco Taxation Directive, which extends the scope of taxation to include e-liquids, heated tobacco, and nicotine pouches. The directive aims to harmonise excise duties across Member States, reduce affordability, particularly for the young, and to close regulatory loopholes that have enabled the expansion of novel nicotine products^1^

This legislative milestone reinforces the urgency of our recommendations: Nicotine in all its forms poses a serious CV threat and calls for unified, cross-product regulatory responses. The following sections discuss each key message, based on epidemiological and mechanistic justifications for a unified nicotine regulation.

## Nicotine-related cardiovascular disease as a leading global health burden

**Figure 1:**
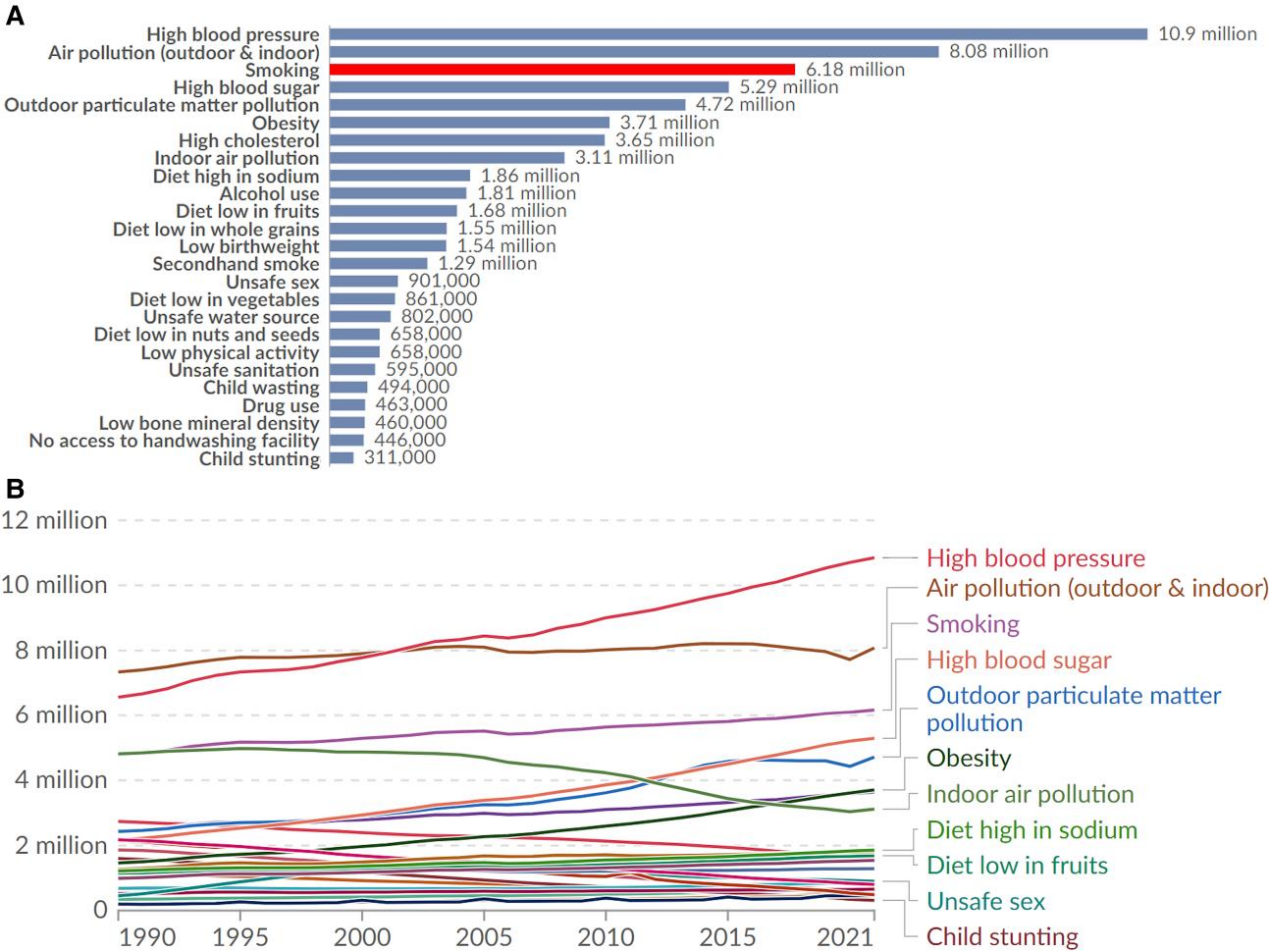
Figure A and B. Global mortality burden of tobacco and other risk factors (Our World in Data, GBD 2021). Panel A shows that in 2021, smoking was the third leading global risk factor for death, responsible for an estimated 6.18 million deaths annually. It surpasses high blood sugar, obesity, and cholesterol, and is only exceeded by high blood pressure (10.9 million) and air pollution (8.08 million). Second-hand smoke contributes an additional 1.29 million deaths, bringing the total tobacco-related burden to over 7.4 million deaths/year, making it one of the most preventable causes of premature mortality. Panel B traces the trajectory of tobacco-related deaths from 1990 to 2021. Smoking-related deaths have remained persistently high, with only a slight upward trend since the mid-2000s, but no evidence of a global decline despite decades of tobacco control. Together, these panels emphasize that tobacco use, both active and passive, continues to be a leading global killer, demanding intensified efforts in tobacco cessation, regulation, and public health education to reduce its cardiovascular and respiratory burden. (adapted with permission from.^2^)

Tobacco smoking and other nicotine-containing products remain among the leading causes of premature mortality worldwide, with CV disease (CVD) as the largest single contributor. According to the Global Burden of Disease (GBD) studies (*Figure 1*):

- Tobacco use accounted for 7.7 million deaths annually, or 13.6% of all global deaths.^3,4^
- It was responsible for nearly 200 million disability-adjusted life years (DALYs) lost worldwide.^4^
- In 2021, smoking alone was responsible for ∼2.25 million CVD deaths globally, mainly from ischaemic heart disease and stroke, confirming CVD as one the leading pathway of tobacco-attributable mortality.^5^
- Globally, smoking also caused an estimated 2.5 million cancer deaths, underscoring that tobacco is both the leading CV *and* oncological risk factor.^3,4^

In Europe alone:

- Tobacco use is responsible for about 1.2 million deaths every year, accounting for 18% of all non-communicable disease deaths in the WHO European Region. Among these, approximately one quarter of CV deaths in men and around 8% in women are attributable to tobacco use.^6^
- The economic cost of smoking in Europe, including healthcare expenditures, productivity loss, and premature mortality, exceeds €300 billion annually^6^

Even more worrisome is that e-cigarettes and new oral nicotine products (ONPs) are accelerating nicotine addiction among the young, especially in high-income countries. Surveys show up to 40% of European adolescents have tried e-cigarettes.^7^ Dual use with cigarettes is now common.

Policy Implication: Nicotine addiction is not declining, it is diversifying. The GBD data show that nicotine-related death and disability remain high. If governments fail to regulate new products, we risk reversing decades of public health gains.

## Nicotine as a direct cardiovascular toxin, regardless of delivery method

**Figure 2:**
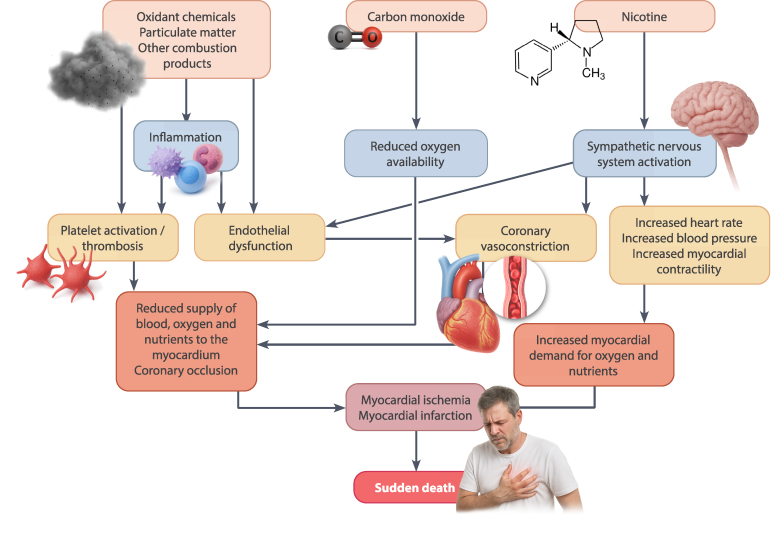
This illustrates the multifaceted CV toxicity of cigarette smoke, highlighting combined effects of ROS, carbon monoxide, and nicotine. Oxidant compounds and particulate matter trigger vascular inflammation, platelet activation, and endothelial dysfunction, central processes that impair vasodilation and promote thrombosis. Carbon monoxide reduces O_2_ availability, exacerbating myocardial hypoxia. Simultaneously, nicotine activates the sympathetic nervous system, leading to increased heart rate, elevated BP, heightened myocardial contractility and also endothelial dysfunction. These haemodynamic changes increase myocardial oxygen demand. Endothelial dysfunction and coronary vasoconstriction compromise coronary blood flow, while platelet activation and thrombosis result in acute coronary occlusion. This mismatch between myocardial O_2_ supply and demand culminates in myocardial ischaemia and infarction, which may lead to sudden cardiac death. Thus, multiple smoke constituents synergistically impair vascular function, destabilize coronary perfusion, and trigger fatal CV events. (adapted with permission from^8^)

Nicotine, the principal alkaloid of the tobacco plant, is often mistakenly considered relatively harmless and addictive, but not causally linked to CVD. This misconception is concerning as growing evidence now identified nicotine itself, independent of combustion, as a potent CV toxin (*Figure 2*). Its effects are biologically active across all delivery systems, e.g. cigarettes, e-cigarettes, heat-not-burn (HNB) devices, waterpipes, and smokeless ONPs.

### Activation of the sympathetic nervous system

Nicotine is a powerful sympathomimetic. It binds to nicotinic acetylcholine receptors in the adrenal medulla and autonomic ganglia, stimulating the release of catecholamines such as norepinephrine and epinephrine. This triggers acute increases in heart rate, myocardial contractility, cardiac output, and peripheral vasoconstriction, all elevating blood pressure (BP) and myocardial oxygen demand.^8^ With chronic exposure, sustained sympathetic activation promotes hypertension, arrhythmias, and cardiac remodelling.^8^

### Vascular dysfunction

Endothelial dysfunction is a well-established early marker of CV risk and atherosclerosis. Nicotine impairs endothelial function through sympathetic activation, reactive oxygen species (ROS), nitric oxide (NO) depletion, inflammatory signalling and BP increases.^9,10^ These effects are seen not only in traditional cigarette smokers but also in users of e-cigarettes and nicotine pouches. Nicotine reduces flow-mediated dilation (FMD)^11^ and increases arterial stiffness in healthy volunteers, even in the absence of other tobacco constituents.^12,13^

In animal models, nicotine leads to endothelial NO synthase (eNOS) uncoupling, increases ROS, and promotes mitochondrial dysfunction.^14^ *In vitro* studies using human endothelial cells show that nicotine upregulates adhesion molecules (e.g. vascular cell adhesion molecule 1 and intracellular adhesion molecule 1, enhancing leukocyte adhesion and vascular inflammation.^10^

### Arterial stiffness and hemodynamic load

Nicotine increases arterial stiffness both acutely and chronically. Increases in pulse wave velocity and augmentation index) reflect increased vascular rigidity following use of cigarettes, e-cigarettes, and ONPs alike.^15–17^ These changes amplify left ventricular afterload and promote heart failure and vascular ageing.

### Cardiac remodelling and fibrosis

Rodent studies demonstrate that nicotine enhances myocardial fibrosis and hypertrophy, resulting in impaired systolic and diastolic function.^18^ These effects occur independently of combustion products and are likely driven by chronic catecholamine stimulation, ROS, and changes in myocardial gene expression.^19^

### Prothrombotic and vasoconstrictive effects

Nicotine also exerts strong prothrombotic effects. It enhances platelet aggregation, increases thromboxane A_2_, and reduces fibrinolysis. Furthermore, it induces coronary vasoconstriction, particularly in segments with pre-existing endothelial dysfunction,^8^ raising the risk of vasospasm and ischaemia in individuals with coronary artery disease.

### Proangiogenic effects

Nicotine exerts potent proangiogenic effects, primarily by activating nicotinic acetylcholine receptors on endothelial cells. Nicotine stimulates endothelial cell proliferation, migration, and capillary tube formation via pathways involving NO, vascular endothelial growth factor, and fibroblast growth factor-2.^1^ This may contribute to plaque neovascularization and instability as well as tumour growth, suggesting that nicotine is not a benign component of tobacco and alternative nicotine products.^19^

### Addiction as a cardiovascular multiplier

The high addictive potential of nicotine ensures repeated, long-term exposure. Adolescents are especially vulnerable to their neurobiological effects, increasing the risk of lifelong use^20^ and becoming a chronic CV burden.

Thus, nicotine is not merely a vector for tobacco addiction; it is itself a biologically active CV toxin acting through well-defined molecular and hemodynamic pathways contributing to all stages of CVD. The policy implications are clear: Regulating nicotine, not just tobacco, is essential to reduce the population with CVD.

## No nicotine-containing product is safe for the heart or blood vessels

The marketing narrative surrounding ‘*safer nicotine products*’ evolved rapidly, from cigarettes to filtered cigarettes, then to e-cigarettes, HNB products, and now to nicotine pouches. Yet despite different delivery platforms and content, scientific evidence confirms that no nicotine-containing product is safe for the CV system. This is now endorsed by the European Society of Cardiology (ESC),^21^ the American Heart Association (AHA),^22^ the U.S. Food and Drug Administration (FDA),^23^ and the World Health Organization (WHO),^24^ and by all organisations together,^25^

### Tobacco cigarettes: the benchmark of harm

Traditional cigarettes remain the most toxic form of nicotine delivery, responsible for the majority of tobacco-attributable deaths worldwide, almost 8 million/year,^3^ Cigarette smoke contains over 9000 chemical compounds, including carbon monoxide (CO), acrolein, benzene, and tobacco-specific nitrosamines (TSNAs).^26,27^ However, even when these combustion by-products are removed, as in nicotine-only products, CV toxicity largely persists, because nicotine itself is harmful,^8,9^

### E-cigarettes: less harmful, not harmless

E-cigarettes have often been marketed as ‘*harm reduction*’ tools. While they emit fewer carcinogens than combusted tobacco, they still expose users to high doses of nicotine, aldehydes (e.g. acrolein, formaldehyde), fine particulate matter, and metal nanoparticles from heating coils.^28,29^ These have been shown to cause endothelial dysfunction, oxidative stress, and arterial stiffness in both animals and humans.^12,30,31^

In FMD studies, e-cigarette users consistently show impaired endothelial function comparable to cigarette smokers.^13,32^ Switching from cigarettes to e-cigarettes may reduce short-term vascular damage,^33^ but chronic use continues to sustain CV risk.^17,34^ Dual use is common and eliminates potential health benefits.^35^

### Heat-not-burn products: cleaner smoke, same threat

HNB devices heat, rather than burn, tobacco. They emit fewer combustion products, reducing exposure to CO and some polycyclic aromatic hydrocarbons (PAHs).^36,37^ However, human and rodent studies show that HNB aerosols still impair endothelial function, and increase ROS and thus reduce vascular NO bioavailability.^38–40^

Switching from cigarettes to HNB products leads to partial vascular recovery, but not normalization.^41^ Moreover, passive exposure to HNB aerosol induces endothelial dysfunction in children, comparable to second-hand cigarette smoke.^42^

### Waterpipes (shisha): misperceived as less harmful

Waterpipe use is often believed to be less harmful due to water ‘filtration’. However, a single 45-minute session delivers as much smoke as 100 cigarettes.^43^ Waterpipe smoke contains CO, TSNAs, heavy metals, and acrolein in concentrations like or exceeding cigarette smoke.^44^ Chronic waterpipe users have impaired FMD, increased arterial stiffness and higher systemic inflammation.^45–47^ Acute waterpipe use, even in passive exposure, causes vascular damage, especially in enclosed spaces with poor ventilation.^48,49^

### Cigars: misperceived safety

Cigar smoking is frequently regarded as less harmful because many users do not actively inhale. However, nicotine and toxic combustion products are readily absorbed through the oral mucosa and upper airways, leading to systemic CV effects. Epidemiological evidence demonstrates that regular cigar use increases the risk of coronary heart disease, stroke, and aortic aneurysm, even among non-inhalers. In 103 642 adults, current cigar use was significantly associated with higher risks of stroke, atrial fibrillation, and heart failure, independent of cigarette smoking.^50^ The perception of ‘*harmless*’ mirrors the misconceptions surrounding waterpipe use and requires explicit correction in public health communication.

### Smokeless oral nicotine products: a new cardiovascular hazard

Nicotine pouches, lozenges, snus, and gums deliver nicotine through the oral mucosa, bypassing combustion. Though they may reduce exposure to inhaled toxicants, they still cause endothelial dysfunction, elevate sympathetic tone, likely impair autonomic regulation and increase BP.^16,22,51^

Recent studies from Sweden link Snus use to higher CV and all-cause mortality, particularly in individuals with prior myocardial infarction or stroke.^52,53^ NHANES and ARIC^54^ confirm increased prevalence of hypertension, peripheral artery disease, and metabolic risk factor clustering among users.^55,56^ Youth-targeted flavours and synthetic nicotine increase the risk of first-time addiction and long-term harm.

### Nicotine Replacement Therapy (NRT): a special case

**Figure 3:**
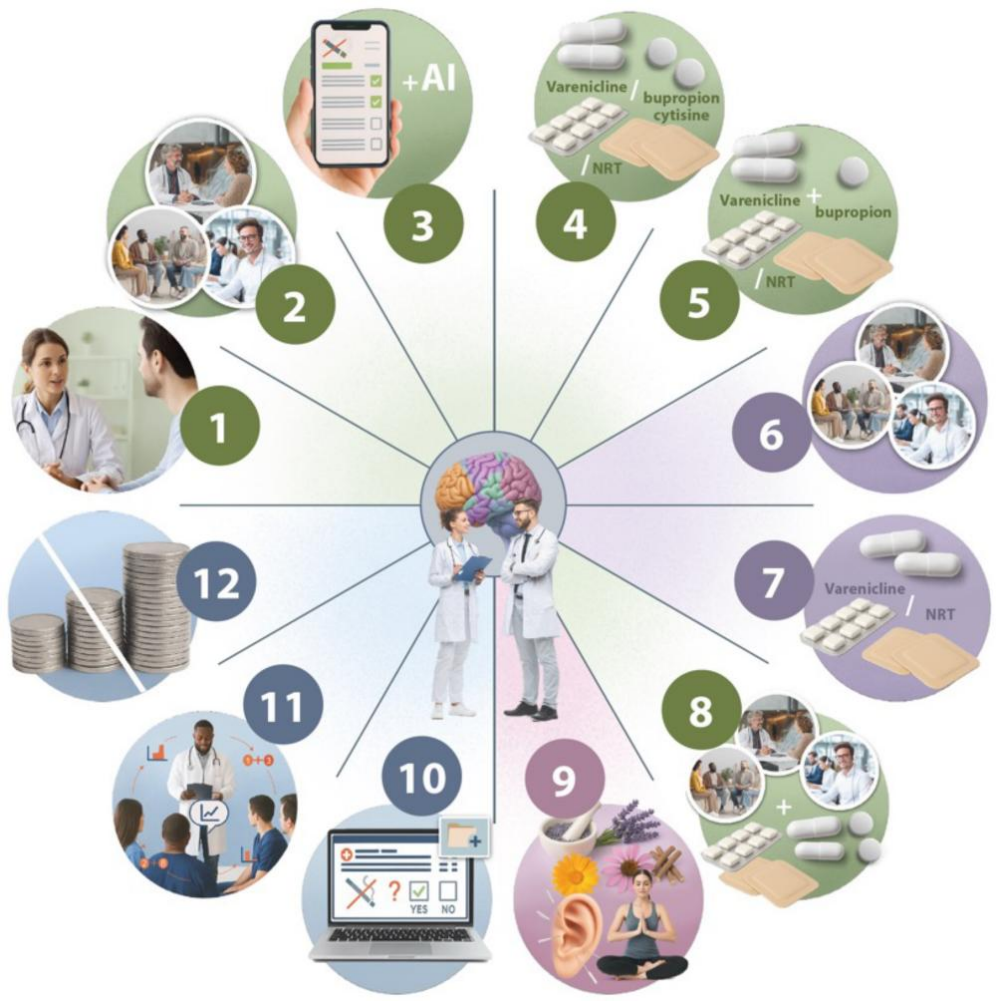
WHO’s 2024 cartoon outlines a comprehensive, evidence-based approach to tobacco cessation centred on clinical engagement. It recommends 12 key strategies to quit. Brief advice (1) should be offered routinely in all healthcare encounters. Intensive behavioural support (2), including individual or group counselling, is advised for those ready to quit. Digital tools like text messages or apps (3) may support cessation, though evidence varies. Pharmacological therapies, varenicline, NRT, bupropion, and cytisine, are first-line options (4), with combination therapies (5) to consider when needed. For smokeless tobacco users, behavioural counselling (6) and varenicline or NRT (7) are also recommended. Combining behavioural and pharmacological support (8) is especially effective. While evidence on alternative therapies (9) is lacking, they may be used alongside established methods. Tobacco use and cessation efforts should be recorded in medical records (10), and all healthcare staff should be trained in cessation delivery (11). Finally, all evidence-based treatments should be available at no or low cost (12). (adapted with permission under the Creative Commons licence CC-BY-NC-SA 3.0 IGO from^57^)

Nicotine replacement therapy is administered in medically supervised, time-limited contexts to support smoking cessation. Although NRT acutely raise BP and heart rate, CV risks seem low and substantially outweighed by the benefits of quitting.^8^ However, NRT is not risk-free and should not be used chronically or outside cessation frameworks. Importantly, the WHO does not recommend e-cigarettes as a cessation strategy (*Figure 3*).^57^

Thus, no form of nicotine use is without CV risk. While non-combusted products may vary in their toxicant profiles, the presence of nicotine is sufficient to impair vascular function, raise BP, and drive atherosclerosis. Policymakers must move beyond combustion as the benchmark of harm and recognize that nicotine itself is harmful.

***Policy takeaway:*** Despite marketing claims of ‘safer’ alternatives, no nicotine-containing product, whether smoked, heated, inhaled, or absorbed, can be considered safe for the heart and blood vessels, a consensus formally supported by the ESC, AHA, FDA, and WHO.^25^ While e-cigarettes, HNB products, waterpipes, and oral nicotine pouches differ in toxicant exposure, all impair endothelial function, elevate BP, and promote atherosclerosis due to nicotine’s inherent CV toxicity. Policymakers must regulate *all* nicotine products, regardless of whether ‘combusted’ or ‘non-combusted’, under a unified CV harm framework.

## E-cigarettes are less harmful than cigarettes, but far from harmless

E-cigarettes have been promoted as a harm reduction tool for adult smokers. Yet the real-world evidence documents sustained addiction, and commonly dual use with continued public health impact. Rather than serving as an exit from smoking, e-cigarettes have become a high-speed on-ramp for youth nicotine addiction.^9^

### A youth epidemic in numbers

Among U.S. high school students, e-cigarette use surged between 2011 and 2019 from 1.5% to 27.5%, surpassing conventional cigarette use.^58^ In the European ESPAD survey, up to 40% of adolescents in certain countries reported having tried e-cigarettes by age 16.^7^ In the UK, Action on Smoking and Health (ASH) has observed similar trends, e.g. there was a 50% growth in experimentation (trying once or twice) from 7.7% in 2022 to 11.6% in 2023 and the number of 11- to 17-year-olds with current vaping has been greater than that of current smoking (7.6% compared with 3.7% in 2023).^59^ The ASH concluded that youth vaping is continuing to grow, as is children’s awareness of promotion. The big increase in the use of disposable products has happened concurrently with higher levels of youth use.^59^ The British Medical Association has called this rapid increase a ‘*public health emergency*’ and urged immediate regulatory action.^60^

Together, these data highlight a cross-continental surge in adolescent nicotine use, underscoring an urgent need for policy intervention. Alarmingly, up to 75% of young adult e-cigarette users (ages 18–20) have never smoked traditional cigarettes.^22^ Thus, e-cigarettes are not replacing cigarettes, but recruiting new users who might otherwise never have used nicotine.

### Addiction by design

Modern e-cigarettes, especially pod-based systems like JUUL, use nicotine salts to deliver high doses of nicotine rapidly and with minimal throat irritation. This pharmacokinetic profile mimics that of combustible cigarettes, promoting deep inhalation and rapid brain delivery.^61^ Nicotine salt formulations are so addictive that even a single pod contains the equivalent of 20 cigarettes.^61^ This has profound implications for adolescent neurodevelopment, with nicotine exposure affecting attention, learning, impulse control, and mood regulation.^20^

### The global vaping surge

**Figure 4:**
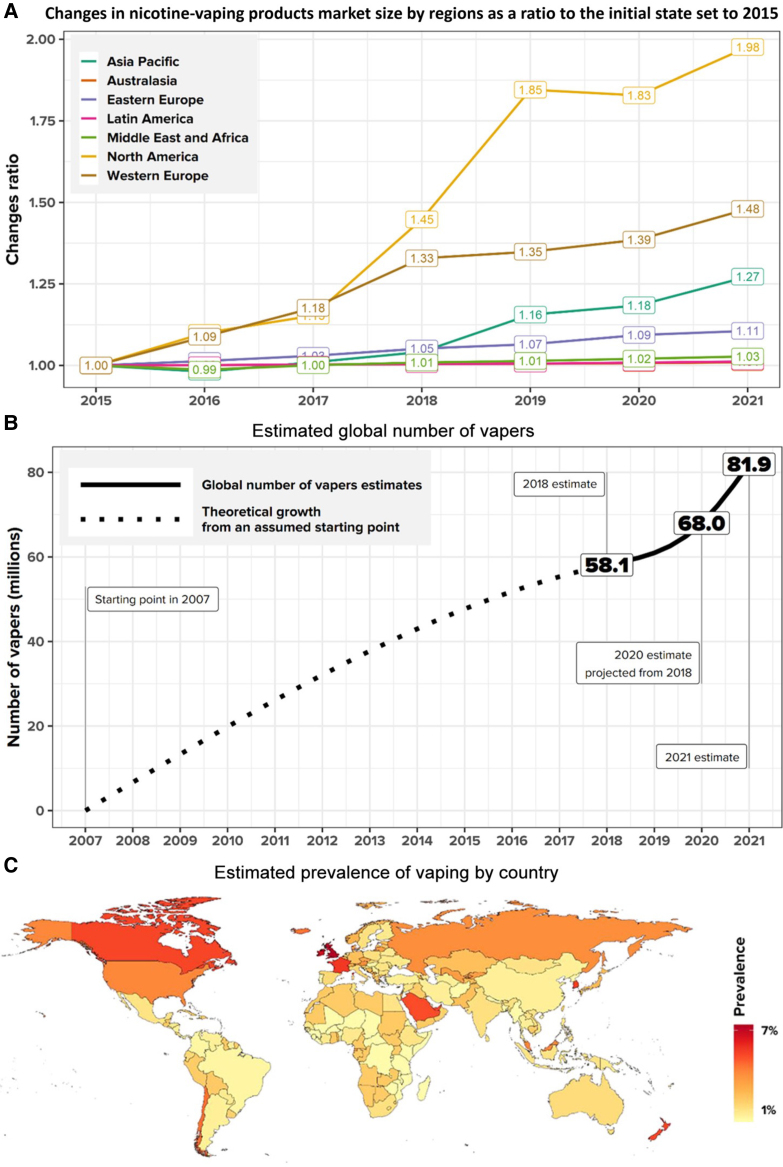
A-C. Global expansion of nicotine-vaping product markets, user numbers, and country-level prevalence. A shows the change in market size of nicotine-vaping products worldwide, as a ratio relative to 2015 levels. By 2021, the market increased 1.98-fold in North America, in Western Europe 1.48-fold and in the Asia Pacific 1.27-fold. Other regions showed slower market expansion. B displays the estimated global number of vapers in 2007–2021. Starting from near-zero in 2007, the number increased steeply, reaching 58.1 million in 2018, 68.0 million in 2020, and 81.9 million by 2021reflecting exponential adoption worldwide. C Estimated prevalence of vaping by country. NA, UK, France, and New Zealand have the highest prevalence (>7%), while most of Africa, South America, and Southeast Asia remain below 1%–3%. This highlights significant geographic disparities in vaping uptake, with high-income countries emerging as key markets. (with permission under the Creative Commons CC-BY 4.0 licence from^62^)

The addiction potential of e-cigarettes within a broader epidemiological and public health framework illustrates in *Figure 4* the rapid rise in global vaping prevalence, regional market expansion, and the steep trajectory of new users, particularly among youth in high-income countries. Due to mechanistic pathways such as oxidative stress, sympathetic activation, and endothelial dysfunction, this underscores that vaping is not just a behavioural concern, but an evolving CV and neurodevelopmental threat on a global scale.

### From vaping to smoking: the gateway pathway

Longitudinal studies have demonstrated that adolescents who start with e-cigarettes are significantly more likely to initiate cigarette smoking within 12–24 months.^63,64^ In fact, e-cigarette use is now a consistent predictor of future combustible tobacco use, even in youth with no prior interest in smoking.

The gateway effect is amplified by social normalization, flavour variety, and the misperception that e-cigarettes are safe or ‘*just water vapour*’. Flavoured products, such as mango, cotton candy, and cola, are especially appealing to the young and are strongly associated with higher initiation rates.^65^

### Dual use: the norm, not the exception

Contrary to their marketed strategy, most e-cigarette users do not fully switch from cigarettes to vaping. Instead, dual use is the dominant pattern, especially in the 18–35 age group.^35^ This dual exposure undermines harm reduction and sustains CV risk from both combustion and nicotine. In line, a recent study in post-myocardial infarction patients established that only those who completely stopped smoking and vaping showed reductions in major adverse cardiovascular events (MACE). Dual users had outcomes similar to smokers^66^

### Limited efficacy as a cessation tool

Evidence for the efficacy of e-cigarettes in smoking cessation is mixed and context-dependent. A Cochrane review reported modest benefit, with about 10%–18% of smokers quitting successfully using e-cigarettes vs 9% with NRT.^67^ However, most quitters continued vaping at 12 months, indicating persistent nicotine dependence.^68^

Importantly, the 2024 WHO guidelines explicitly do not recommend e-cigarettes as a cessation tool, due to insufficient long-term safety data.^57^ The ESC and AHA adopt a similar position and suggest that e-cigarettes should be considered only as a last resort, within structured cessation programmes and not as over-the-counter consumer products.^21,22^

### How quitting, switching to e-cigarettes, or sticking to smoking shapes cardiovascular outcomes after percutaneous coronary intervention

**Figure 5:**
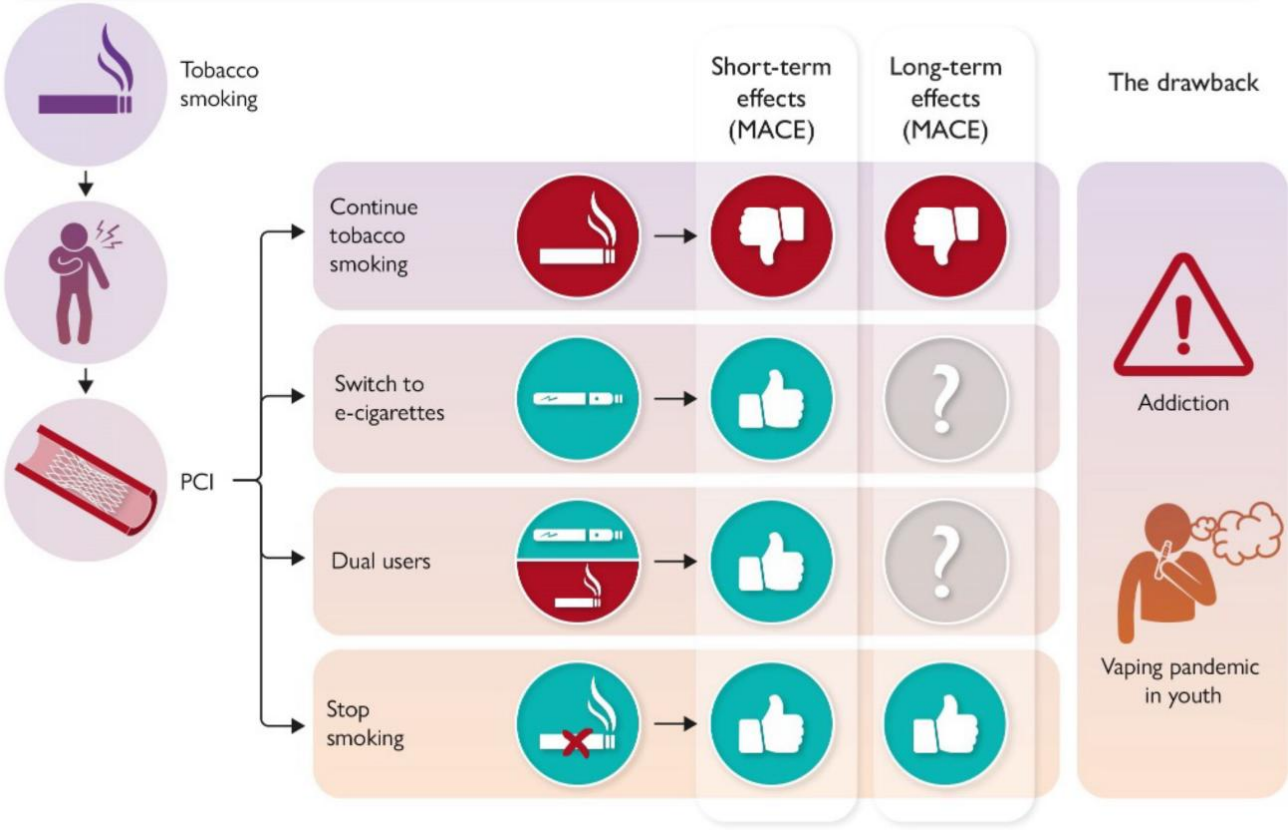
The potential CV outcomes after PCI for tobacco-related CVD. Continuing smoking leads to negative short- and long-term impacts. Switching to e-cigarettes or dual use shows positive short-term effects, but uncertain long-term outcomes. Stopping smoking entirely results in positive outcomes both short- and long-term. Additionally, the diagram highlights the other side of the coin concerning vaping addiction, especially among youth. (with permission from^69^)

Studies investigating CV outcomes following a switch from tobacco cigarettes to e-cigarettes remain scarce. A recent large-scale study of over 33 000 patients after percutaneous coronary intervention (PCI) compared persistent smokers, complete quitters, exclusive e-cigarette users, and dual users.^66^ As expected, smoking cessation conferred the greatest reduction in MACE. Those who switched to e-cigarettes experienced a modest risk reduction, whereas dual users derived no benefit. While short-term vascular improvements have been observed with e-cigarettes, the absence of long-term safety data, especially amid escalating youth addiction, remains a major public health concern^66^ (*Figure 5*).

### Regulatory responses and gaps

In response to mounting evidence of youth harm, numerous countries have banned flavoured e-liquids, restricted social media marketing, or raised the minimum legal purchase age. In 2022, the U.S. FDA issued marketing denial orders for several flavoured e-cigarette products, including JUUL.^23^ However, enforcement gaps and online availability erode these measures.

In contrast, the UK continues to promote e-cigarettes as harm reduction tools via the National Health Service, despite growing calls for a more precautionary approach.^70^

**Policy takeaway:** E-cigarettes have failed to fulfil their promise in smoking cessation, while fuelling a youth addiction crisis. Nicotine dependence has shifted, not disappeared. These products must be subject to the same advertising bans, indoor-use restrictions, and taxation policies as combustible tobacco to prevent another generation from lifelong CV harm.

## Passive nicotine exposure: an invisible cardiovascular risk

Nicotine policies primarily centre on active users. Yet non-users are not spared from passive exposure. Inhaling smoke, aerosol, or vapour second-hand, whether from cigarettes, e-cigarettes, waterpipes, or heated tobacco products, can cause measurable endothelial dysfunction in minutes. Passive nicotine exposure is a silent but potent driver of CVD and should be a regulatory priority.

### Second-hand smoke: a well-documented killer

According to the WHO, over 1.2 million deaths/year are attributable to second-hand tobacco smoke exposure alone.^24^ Non-smokers living with smokers have a 30% increased risk of coronary artery disease, even after adjusting for other risk factors.^71^ Exposure of less than 30 min, acutely impair endothelial function and increase platelet aggregation, triggering myocardial infarction or stroke in vulnerable individuals.^10,72^

**Figure 6:**
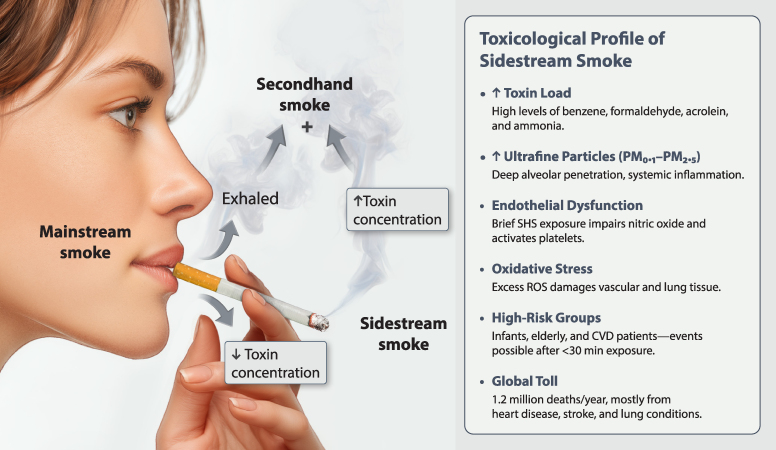
Toxicological comparison of mainstream and sidestream smoke. While mainstream smoke is inhaled directly by the smoker and contains a lower toxin concentration, sidestream smoke (a major component of passive smoking) carries a higher concentration of toxicants, including ultrafine particles and reactive chemicals. This leads to ROS, endothelial dysfunction and CVD. Vulnerable groups, such as infants, the elderly, and patients with CVD, are at risk even after brief exposure. Sidestream smoke contributes to over 1.2 million deaths/year worldwide

Sidestream smoke, emitted from the burning end of a cigarette, contains higher concentrations of toxicants than the mainstream smoke inhaled by smokers (*Figure 6*). This includes ammonia, benzene, formaldehyde, acrolein, and fine particulate matter (PM2.5).^9^ Sidestream smoke is also richer in ultrafine particles, which more easily penetrate the alveolar membrane and reach systemic circulation.^73^

### E-cigarette aerosols: not just harmless vapour

The widespread perception that e-cigarette aerosols are ‘*just water vapour*’ is incorrect. Passive exposure to exhaled vapour, particularly in enclosed environments, introduces nicotine, propylene glycol, aldehydes (e.g. acrolein, formaldehyde), metals, and ultrafine liquid particles into the ambient air.^31,74^

Even short-term exposure to e-cigarette aerosol in animal and human studies induces ROS, inflammation and endothelial dysfunction, comparable to passive cigarette smoke.^30,75^ A recent study found that non-smokers exposed to exhaled e-cigarette aerosols showed impaired FMD and elevated markers of vascular inflammation.^13^

As a result, both the ESC and WHO now recommend banning e-cigarette use in public indoor spaces, alongside traditional smoking, to protect bystanders.^76,77^

### Waterpipes: a hidden threat in communal settings

Waterpipe sessions produce dense smoke clouds that easily spread through homes, cafes, and lounges. The burning charcoal used for heating tobacco generates high levels of CO, PAHs, PM_2.5_, and benzene. Passive exposure can exceed that of cigarettes, one session may equal the smoke volume of over 100 cigarettes.^43,78^

Passive exposure to waterpipe smoke causes acute endothelial dysfunction and impairs vascular reactivity in healthy bystanders.^48^ Children and pregnant women are particularly at risk, and yet many waterpipe venues lack ventilation regulations or smoke-free zones.^49^

### Heated tobacco products: still a risk to bystanders

While HNB devices release fewer visible emissions, the aerosol still contains nicotine, aldehydes, volatile organic compounds, and ultrafine PM, all of which can affect non-users. Even passive exposure to HNB aerosol impairs FMD and increases oxidative stress markers in children and young adults.^42,79^

Because aerosols are often odourless and dissipate quickly, bystanders may not even be aware of their exposure, creating an invisible but real CV risk.

### Smokeless nicotine products: minimal airborne risk, surface contamination concerns

Smokeless oral nicotine products (ONPs), such as snus, nicotine pouches, lozenges, and gums, do not emit aerosols or sidestream vapours because they do not involve combustion or heating. Thus the risk of airborne passive exposure is negligible.

**Policy Implication:** Indoor air quality must be protected from *all* forms of nicotine delivery. Policymakers should extend smoke-free laws to cover e-cigarettes, heated tobacco, and waterpipe use in public and private enclosed spaces. Public messaging must refrain of the ‘*clean vapour*’ myth and educate citizens on the vascular risks of passive nicotine exposure.

## The vascular red flag: how nicotine disrupts endothelial health across all products

The vascular endothelium serves as a gatekeeper for CV health. It regulates blood flow, inflammation, platelet activity, and vascular tone. When it becomes dysfunctional, characterized by reduced NO bioavailability and increased ROS, setting the stage for atherosclerosis, thrombosis, and ultimately MACE. Nicotine, regardless of its delivery system, has been shown to impair endothelial function consistently and rapidly.

This makes endothelial dysfunction an ideal early biomarker of nicotine-induced vascular damage, a scientific insight with direct policy relevance.

### A central mechanism across all nicotine products

**The harmful CV effects of nicotine have been demonstrated in:**

- Cigarette smokers: Chronic and even single-cigarette exposure significantly reduces FMD.^80,81^
- E-cigarette users: Acute and chronic use impairs FMD and microvascular reactivity.^12,32^
- Waterpipe smokers: FMD is markedly reduced even after a single session.^45,46^
- Heated tobacco and HNB users: Comparable endothelial impairment has been documented.^38,39^
- Smokeless oral nicotine users: Lower FMD and increased arterial stiffness reported in both acute and chronic settings.^16,82^

### Oxidative stress and endothelial nitric oxide synthase uncoupling

The molecular mechanism behind nicotine-induced endothelial dysfunction centres on ROS and the uncoupling of eNOS. Under normal conditions, eNOS produces protective NO. In nicotine-exposed individuals, the enzyme becomes dysfunctional, generating O_2_^-^ instead of NO, amplifying oxidative damage.^75,83^ Indeed, antioxidant treatment (e.g. vitamin C, tetrahydrobiopterin) acutely improves endothelial function in smokers, confirming the role of oxidative stress as a reversible contributor.^81^

### Endothelial dysfunction predicts events

Endothelial dysfunction is not merely a mechanistic curiosity, it is a validated predictor of MACE, including myocardial infarction and stroke.^84^ The degree of dysfunction correlates with smoking intensity and duration, and recovery is only partial even after cessation. This underscores why even occasional, or low-dose nicotine exposure cannot be considered safe, particularly in individuals with pre-existing CVD.

### Animal and cellular confirmation

Nicotine-containing aerosol exposure (e.g. from e-cigarettes or HNB) induces oxidative stress, nicotinamide adenine dinucleotide phosphate oxidase 2 (NOX-2 activation), and eNOS uncoupling. FMD is impaired *in vivo*, and similarly is vascular reactivity in isolated aortic rings.^12,85,86^ Cell culture models add further confirmation: When endothelial cells are incubated with sera from e-cigarette or cigarette users, NO release is sharply reduced, and ROS increase substantially.^13,32^

Together, these findings clarify that nicotine, through oxidative injury, causes vascular dysfunction across all product types and species, providing a mechanistic basis for regulatory harmonization.

**Policy takeaway:** Endothelial dysfunction offers policymakers a unique opportunity: a sensitive and early, non-invasive biomarker that reveals vascular toxicity of emerging nicotine products long before clinical disease manifests. Regulators should treat any nicotine product that impairs endothelial function as inherently unsafe for CV health.

## The hidden bill: how nicotine harms hearts, and bleeds public budgets

The harm caused by nicotine is not confined to endothelial cells or laboratory conditions. It manifests itself as CVD, such as stroke, heart failure, and early death. But beyond the clinical toll lies an enormous economic public burden for healthcare systems, employers, and society at large. Policymakers must reckon with nicotine not only as a biochemical hazard, but as an economic liability.

### Cardiovascular disease: the leading cause of tobacco-attributable death

Globally, tobacco use is responsible for almost 8 million deaths/year, of which more than half are CV in origin.^24^ The GBD 2019 study ranked high systolic BP and tobacco use among the top global risk factors for death and DALYs.^3,4^

A systematic analysis of 52 countries (INTERHEART) showed that current smokers had a 2.95-fold higher risk of acute myocardial infarction compared with never smokers.^87^ These effects are dose-dependent and begin even at low exposure. Similar results have been replicated for stroke, heart failure, peripheral artery disease, and sudden cardiac death.^88,89^

### Emerging nicotine products also raise cardiovascular risk

Contrary to industry messaging, alternative nicotine delivery systems are not benign. Evidence now implicates e-cigarettes, HNB products, and smokeless nicotine products in a broad spectrum of MACE:

- E-cigarettes: Associated with increased risk of myocardial infarction (odds ratio up to 2.1).^90,91^
- Waterpipes: Linked to coronary artery stenosis and ischaemic heart disease.^92,93^
- Snus and ONPs: Associated with increased post-infarction mortality and progression of atherosclerosis.^52,53^

While relative risk may be lower than for cigarettes, absolute risk remains substantial, especially in patients with underlying CVD.

### The economic cost: a heavy burden on public systems

The WHO estimated global economic cost of tobacco use at over USD 1.4 trillion/year, amounting to 1.8% of the world’s annual GDP. CV care accounts for a significant proportion of these costs, given the need for long-term treatment, medications, PCI, hospitalisations, and rehabilitation. In the EU alone, tobacco-related healthcare costs exceed €25 billion/year, with additional losses in productivity from disability and premature death, estimated at €8.3 billion. E-cigarettes and newer products, while often excluded from tobacco cost models, are expected to contribute increasingly to future CVD-related expenditures, particularly through:

- Higher incidence of hypertension and arrhythmias in the youth.
- Increased use of cardiac imaging, interventions, and chronic pharmacotherapy.
- Growing incidence of dual use, compounding exposure and cost.

### Disproportionate impact on low- and middle-income countries

Over 80% of the world’s 1.3 billion tobacco users now live in low- and middle-income countries (LMICs).^24^ These nations are often targeted aggressively by tobacco and vaping companies and have weaker healthcare systems, less regulatory oversight, and limited access to CV care. The result is a vicious cycle of addiction, CVD, and economic stagnation. Unlike high-income countries, where the cost burden is partially absorbed by insurance and health infrastructure, LMICs absorb the cost directly through lost productivity and avoidable mortality.

**Policy takeaway:** Nicotine-containing products are not just a health hazard, they are an economic drain. Their continued use fuels hospital admissions, burdens healthcare budgets, and undermines national productivity. Policymakers must treat nicotine regulation as fiscal policy. Banning flavoured products, raising excise taxes, and closing regulatory loopholes will yield both health and economic dividends.

## The next generation hooked: how the nicotine industry rebrands addiction for teens

Nicotine addiction is increasingly seeded not in adulthood, but in adolescence. Today’s adolescents are exposed to an unprecedented array of flavoured, sleek, and socially marketed nicotine products, many of which are incorrectly perceived as safe. Behind this lies a powerful industry that aggressively markets addiction under the guise of ‘*harm reduction*’. Policymakers must treat this as an urgent public health emergency.

### An explosion of youth nicotine use

Across Europe and North America, youth nicotine use has skyrocketed:

- In the USA, e-cigarette use among high school students rose from 1.5% in 2011 to 27.5% in 2019, making it the most used tobacco product among the young.^74^
- In Germany, 38% of adolescents aged 14–17 have tried e-cigarettes, and over 60% of these users prefer flavoured variants.^94^
- The 2019 ESPAD survey across 35 European countries found that over 40% of 15–16-year-olds had tried an e-cigarette, often as a first exposure to nicotine.^7^

More worryingly, three-quarters of young adult e-cigarette users (aged 18–20) in Europe have never smoked conventional cigarettes, a reversal of the cessation narrative and evidence that these products are creating new addicts.^22,95^

### Industry playbook: flavours, influencers, and ‘harm reduction’

Nicotine companies use strategies eerily reminiscent of the 20th-century tobacco playbook:

- Flavours like cotton candy, mango, and mint make products more palatable and disguise the harshness of nicotine.^24^
- Social media influencers, many with millions of adolescent followers, promote vaping devices as lifestyle accessories.^96^
- ‘*Tobacco-free*’ labelling misleads consumers into believing that ONPs are safer, when they are equally addictive.^97^

The goal is simple: rebrand nicotine as a wellness product. In doing so, the industry sidesteps decades of anti-tobacco regulation, targeting the most vulnerable population, youth, before regulatory frameworks can respond.

### Neurobiological vulnerability in adolescence

The adolescent brain is highly susceptible to nicotine-induced neuroadaptations. Nicotine exposure during this critical developmental window:

- Alters dopamine signalling, enhancing reward sensitivity and addiction potential.^8^
- Primes the brain for other substance use, as shown in longitudinal studies linking adolescent vaping to subsequent smoking and cannabis use.^20^
- Increases lifetime risk of impulse disorders, mood instability, and addiction persistence.^98^

In effect, early nicotine exposure locks young people into a lifelong health and economic disadvantage.

### The failure of current youth protections

Despite declarations of youth protection, flavoured products remain widely available in most EU countries, often online and with minimal age verification.

- In France, over 90% of 16-year-olds can access nicotine pouches and e-cigarettes online.
- Only a minority of EU countries have banned flavours or implemented plain packaging for non-cigarette nicotine products.

Moreover, taxation and legal age limits are inconsistent, with synthetic nicotine often escaping tobacco regulations entirely.

**Policy takeaway:** Nicotine addiction is being reshaped as a youth epidemic, enabled by aggressive marketing and regulatory loopholes. Governments must act decisively: ban flavours, enforce plain packaging, close synthetic nicotine loopholes, raise the minimum age for purchasing all nicotine products, and extend smoke-free laws to cover all products. Without urgent action, the next generation will be the most addicted since the cigarette era began.

## One message, one risk: no safe nicotine for the cardiovascular system

Across decades of research and shifting product landscapes, one truth has emerged with growing clarity: there is no safe form of nicotine for the CV system. From conventional tobacco cigarettes to modern devices like e-cigarettes, heated tobacco, and oral pouches, the effects of nicotine on the vasculature are consistent, harmful, predictable, and preventable.

### Endothelial dysfunction: a shared outcome

The vascular endothelium serves as a gatekeeper for CV health. It regulates blood flow, inflammation, platelet activity, and vascular tone. When it becomes dysfunctional, characterized by reduced NO bioavailability and increased ROS, it sets the stage for atherosclerosis, thrombosis, and ultimately MACE. Nicotine, regardless of its delivery system, has been shown to impair endothelial function consistently and rapidly. This makes endothelial dysfunction an ideal early biomarker of nicotine-induced vascular damage, a scientific insight with direct policy relevance.

### A central mechanism across all nicotine products

Experimental and clinical studies confirm that nicotine activates the sympathetic nervous system, increases heart rate and BP, induces oxidative stress, and reduces NO availability. These effects converge to impair FMD, a well-established marker of endothelial function.^10,99^

**This has been demonstrated in:**

- Cigarette smokers: Chronic and even single-cigarette exposure significantly reduces FMD.^80,81^
- E-cigarette users: Acute and chronic use impairs FMD and microvascular reactivity.^12,32^
- Waterpipe smokers: FMD is markedly reduced after a single session.^45,46^
- Heated tobacco and HNB users: Comparable endothelial impairment has been documented.^38,39^
- Smokeless oral nicotine users: Lower FMD and increased arterial stiffness reported in both acute and chronic settings.^16,82^

### Scientific societies speak with one voice

Major CV and public health organisations now align behind a clear stance:

- The ESC stated in 2024 that ‘no form of nicotine use is safe for cardiovascular health,’ and called for inclusion of all nicotine delivery systems in indoor smoking bans and taxation frameworks.^21,76^
- The AHA warns that ‘*smokeless and electronic nicotine products present novel cardiovascular risks, particularly among youth and dual users*’.^22^
- The WHO reiterated in its 2024 technical note that ‘*there is no public health basis for classifying any nicotine product as safe or approved for cessation*,’ urging member states to regulate or ban novel nicotine products.^77^
- In 2021, the *WHO, AHA, ACC, and ESC* issued a joint ‘tobacco endgame’ statement, calling for accelerated global action to eliminate tobacco use as a driver of CVD and premature mortality^25^
- In their recent 2025 report, the European Environment Agency (EEA) firmly rejects the tobacco industry’s ‘*harm reduction*’ narrative. Under the EU’s *Zero Pollution and Health Protection* agenda, it stresses that no industry can claim to reduce harm while selling products that damage health and the environment.^100^ Tobacco and nicotine products, including e-cigarettes and heated tobacco, are identified as sources of preventable pollution and disease, not instruments of health protection. The EEA warns that the industry’s so-called harm reduction strategies divert attention from proven measures such as taxation, advertising bans, and smoking cessation.^100^
- This consensus extends to multiple clinical guidelines, which have removed e-cigarettes from smoking cessation recommendations, underscoring that their long-term safety is unproven and their short-term harms are already visible.

### From pharmacology to public health

The CV effects of nicotine are not dose-dependent in the way many policymakers assume. Even occasional or low-dose use:

- Increases heart rate and arterial stiffness.^101^
- Promotes endothelial dysfunction and platelet activation.^13^
- Can trigger ischaemia in vulnerable individuals.^33^

These effects are compounded by poly-use, which is increasingly common, e.g. dual use of e-cigarettes and pouches, or waterpipe and cigarettes. This shifts the burden from individual behaviour to population-level exposure and cost.

**Policy Takeaway:** The CV evidence is unambiguous: no nicotine product is safe. A unified message from science must translate into a unified policy that rejects industry narratives of harm reduction in favour of explicit, enforceable protections against all nicotine forms. Regulation must be risk-based, not market-driven. In addition, no more exceptions in legislature to tobacco use (i.e. Snus in Sweden).

## From loopholes to leadership: turning cardiovascular science into binding nicotine policy

Scientific consensus alone is not enough. To protect CV health at the population level, evidence must be translated into binding regulation. And to defend the next generation, policymakers must stop allowing the nicotine industry to outpace public health law. Countries that implement comprehensive nicotine regulation now are likely to achieve long-term benefits in population health, workforce productivity, and economic sustainability.

### Tobacco control works—if applied without loopholes

The WHO Framework Convention on Tobacco Control, ratified by 181 countries, remains the global gold standard. Its pillars, taxation, advertising bans, health warnings, smoke-free laws, and cessation services, reduced smoking rates and CV mortality;^98^ however, these pillars were designed for cigarettes, and many countries have failed to apply them to new nicotine products.

**In Europe:**

- Snus is banned in most EU countries, but synthetic oral pouches are legal and unregulated in many of the same states.
- E-cigarette flavours remain available in over 25 EU nations, even as youth uptake surges.^7^
- HNB products are taxed significantly lower than conventional cigarettes in 21 EU countries.^102^

These inconsistencies weaken the credibility of public health policy and leave the door wide open for the nicotine industry to exploit regulatory loopholes and attract new users.

### Recommended legislative actions

To address these shortcomings and bring nicotine regulation in line with current CV science, we propose the following legislative measures:

1. Ban Flavoured Products: All nicotine-containing products with flavouring agents, especially fruit, mint, and candy variants, should be prohibited. Flavours are a major driver of youth initiation and undermine cessation efforts.^20,24^
2. Tax All Nicotine Products Equally and raise taxation gradually. Create a unified excise tax structure based on nicotine content and risk profile. The principle: more nicotine, more tax, regardless of delivery method. Differential taxation incentivises dual use and industry circumvention.
3. Mandate Plain Packaging: Extend plain packaging requirements to e-cigarettes, ONPs, and HNB devices. Branding, colours, and shape modifications are major marketing tools used to attract youth.^97^
4. Close Online Sales and Advertising Loopholes: Enforce age verification and restrict all online sales of nicotine products. Ban marketing via social media and influencer platforms. Device-only advertising, which HNB brands use to sidestep tobacco laws, should also be prohibited.^103^
5. Expand indoor air laws to cover e-cigarettes, HNBs, and waterpipes. Exhaled aerosol contains fine particles, aldehydes, and metals harmful to bystanders. These protections should also extend to outdoor terraces, cafés, and similar semi-enclosed public spaces, where exposure can be substantial.^34,74^
6. National Cardiovascular Prevention Plans Must Address Nicotine: Countries must integrate nicotine harm into non-communicable disease strategies, CV action plans, and health cost modelling. A tobacco-free generation requires intersectoral planning, not siloed regulation.
7. Redirecting public funds away from tobacco subsidies and towards health promotion is essential to protect CV health and ensure responsible use of taxpayers’ money.
8. Ban Tobacco Product Placement in Media and Entertainment: The continued visibility of tobacco and nicotine products in movies, streaming platforms, and social media perpetuates the normalization of smoking and vaping. Product placement, often showing actors smoking in close-up, serves as indirect advertising, particularly influential among adolescents and young adults. To counter this, strict bans on tobacco imagery and sponsorship in films, television, and digital media are essential, accompanied by mandatory anti-tobacco disclaimers where historical depictions are unavoidable.

### The role of health ministries and scientific societies

Regulation alone is insufficient. Ministries of Health must:

- Invest in cessation services, especially in primary care, cardiology, and pulmonology.
- Fund public education campaigns highlighting nicotine’s CV harms.
- Monitor emerging products—including synthetic nicotine variants—for toxicity and marketing practices.

Scientific societies (e.g. ESC, ACC, AHA) must act as independent watchdogs, resisting industry attempts to rebrand nicotine as therapeutic. Their guidelines must continue to exclude all nicotine products from CV prevention frameworks unless they are proven safe, which none currently are.

**Policy takeaway:** The inconsistent regulation of nicotine products is not due to lack of evidence but to lack of political resolve. A uniform framework, covering taxation, flavour bans, plain packaging, advertising restrictions, smoke-free laws, and cessation support, is urgently needed. Applying these proven measures to all nicotine delivery systems will reduce CVD, protect youth, and strengthen the integrity of public health policy against industry influence.

## Fighting passive exposure: the case for total smoke-free spaces

The CV effects of passive nicotine exposure are now unequivocally established. Even brief exposure to second-hand smoke or exhaled e-cigarette aerosol can impair endothelial function, raise oxidative stress, and increase thrombogenicity in healthy non-smokers.^9,31^ Children, pregnant women, and patients with cardiovascular disease are particularly vulnerable.

To protect bystanders, especially in densely populated urban environments, regulatory action must extend beyond indoor bans. The following measures are essential:

- Prohibit smoking and vaping in all outdoor areas where children or vulnerable populations are present, including:
  - Playgrounds, schools, and recreational zones.
  - Outdoor seating areas of restaurants, cafes, and bars.
  - Public transport stops, platforms, and stations.
  - Hospital and healthcare facility grounds.
  - Parks and sports stadiums.
- Introduce visible signage and enforceable fines for violations, mirroring successful models from countries like France and New Zealand, where bans on outdoor smoking near schools and playgrounds are already implemented.
- Include waterpipes and e-cigarettes in all smoke- and aerosol-free regulations, due to their proven potential to harm bystanders through CO, aldehydes, ultrafine particles, and nicotine residue.^24,46^
- Monitor air quality in high-traffic public venues to detect and document second-hand aerosol pollution, especially in semi-enclosed spaces like train stations, stadiums, or covered restaurant terraces.

**Policy takeaway:** Every citizen deserves clean air, regardless of where they sit, eat, or play. Expanding smoke-free laws to outdoor public spaces is not only a health intervention, it is a declaration of public dignity and equity.

## A call to action for cardiologists and policymakers

The evidence is unequivocal: nicotine, across all delivery systems, is a direct cardiovascular toxin.^8,9,32,81^ Its CV side effects are consistently demonstrated in mechanistic studies, clinical trials, and epidemiology. The damage begins early, persists beyond cessation, and is amplified by poly-use of products.

For *cardiologists*, nicotine prevention must become a standard part of CV prevention, just like hypertension or lipid management. Every consultation should include nicotine screening and cessation support, with integration into guidelines, professional training, and reimbursement for pharmacotherapy and counselling.

For *policymakers*, the mandate is equally clear. Protecting future generations requires banning flavoured and synthetic nicotine products, enforcing plain packaging and advertising restrictions, expanding smoke-free laws indoors and outdoors, ensuring taxation proportional to harm, and funding robust cessation services.

This is not a lifestyle issue but a structural determinant of CV health. Although our focus here is Europe, the implications are global, especially in LMICs where nicotine use is rising under weak regulation. Every day of delay costs lives, fuels addiction, and widens inequalities.

Thus, we call on governments, ministries, parliaments, and supranational agencies to act decisively, not cautiously. The CV evidence leaves no room for compromise. The next infarction, the next stroke, and the subsequent death may not come from a cigarette but from a sleek pod, a flavoured pouch, or a waterpipe in a café.

## Declarations

### Data Availability

No data were generated or analysed for or in support of this paper.

## References

[1] European Commission. European Commission modernises Tobacco Taxation Directive. https://taxation-customs.ec.europa.eu/news/european-commission-modernises-tobacco-taxation-directive-2025-07-16_en#:∼:text=Extending the scope of the,with the Better Regulation principles (19 July 2025, date last accessed).

[2] (IHME) IfHMaE. 2021. Global Burden of Disease—Risk Factors. https://ourworldindata.org/grapher/number-of-deaths-by-risk-factor?tab=line&time=earliest (20 July 2025, date last accessed).

[3] Murray CJL, Aravkin AY, Zheng P, Abbafati C, Abbas KM, Abbasi-Kangevari M, et al Global burden of 87 risk factors in 204 countries and territories, 1990–2019: a systematic analysis for the Global Burden of Disease Study 2019. Lancet 2020;396:1223–49. 10.1016/S0140-6736(20)30752-2 33069327 PMC7566194

[4] Reitsma MB, Kendrick PJ, Ababneh E, Abbafati C, Abbasi-Kangevari M, Abdoli A, et al Spatial, temporal, and demographic patterns in prevalence of smoking tobacco use and attributable disease burden in 204 countries and territories, 1990–2019: a systematic analysis from the Global Burden of Disease Study 2019. Lancet 2021;397:2337–60. 10.1016/S0140-6736(21)01169-7 34051883 PMC8223261

[5] Zhu S, Gao J, Zhang L, Dong W, Shi W, Guo H, et al Global, regional, and national cardiovascular disease burden attributable to smoking from 1990 to 2021: findings from the GBD 2021 Study. Tob Induc Dis 2025;23:1-11. 10.18332/tid/200072 PMC11784507 39897459

[6] WHO. Status of tobacco use in the Region. https://www.who.int/europe/news-room/fact-sheets/item/tobacco (29 September 2025, date last accessed).

[7] The ESPAD Group. ESPAD Report 2019: Results from the European School Survey Project on Alcohol and Other Drugs. https://www.espad.org/espad-report-2019 (4 September 2025, date last accessed).

[8] Benowitz NL, Burbank AD. Cardiovascular toxicity of nicotine: implications for electronic cigarette use. Trends Cardiovasc Med 2016;26:515–23. 10.1016/j.tcm.2016.03.001 27079891 PMC4958544

[9] Munzel T, Hahad O, Kuntic M, Keaney JF, Deanfield JE, Daiber A. Effects of tobacco cigarettes, e-cigarettes, and waterpipe smoking on endothelial function and clinical outcomes. Eur Heart J 2020;41:4057–70. 10.1093/eurheartj/ehaa460 32585699 PMC7454514

[10] Heiss C, Amabile N, Lee AC, Real WM, Schick SF, Lao D, et al Brief secondhand smoke exposure depresses endothelial progenitor cells activity and endothelial function: sustained vascular injury and blunted nitric oxide production. J Am Coll Cardiol 2008;51:1760–71. 10.1016/j.jacc.2008.01.040 18452782

[11] Neunteufl T, Heher S, Kostner K, Mitulovic G, Lehr S, Khoschsorur G, et al Contribution of nicotine to acute endothelial dysfunction in long-term smokers. J Am Coll Cardiol 2002;39:251–6. 10.1016/S0735-1097(01)01736-7 11788216

[12] Kuntic M, Oelze M, Steven S, Kroller-Schon S, Stamm P, Kalinovic S, et al Short-term e-cigarette vapour exposure causes vascular oxidative stress and dysfunction: evidence for a close connection to brain damage and a key role of the phagocytic NADPH oxidase (NOX-2). Eur Heart J 2020;41:2472–83. 10.1093/eurheartj/ehz772 31715629 PMC7340357

[13] Carnevale R, Sciarretta S, Violi F, Nocella C, Loffredo L, Perri L, et al Acute impact of tobacco vs electronic cigarette smoking on oxidative stress and vascular function. Chest 2016;150:606–12. 10.1016/j.chest.2016.04.012 27108682

[14] Dikalov S, Itani H, Richmond B, Vergeade A, Rahman SMJ, Boutaud O, et al Tobacco smoking induces cardiovascular mitochondrial oxidative stress, promotes endothelial dysfunction, and enhances hypertension. Am J Physiol Heart Circ Physiol 2019;316:H639–46. 10.1152/ajpheart.00595.2018 30608177 PMC6459311

[15] Vlachopoulos C, Ioakeimidis N, Abdelrasoul M, Terentes-Printzios D, Georgakopoulos C, Pietri P, et al Electronic cigarette smoking increases aortic stiffness and blood pressure in young smokers. J Am Coll Cardiol 2016;67:2802–3. 10.1016/j.jacc.2016.03.569 27282901

[16] Antoniewicz L, Kabele M, Nilsson U, Pourazar J, Rankin G, Bosson JA, et al Chronic snus use in healthy males alters endothelial function and increases arterial stiffness. PLoS One 2022;17:e0268746. 10.1371/journal.pone.0268746 35657943 PMC9165771

[17] Lyytinen G, Melnikov G, Brynedal A, Anesäter E, Antoniewicz L, Blomberg A, et al Use of heated tobacco products (IQOS) causes an acute increase in arterial stiffness and platelet thrombus formation. Atherosclerosis 2024;390:117335. 10.1016/j.atherosclerosis.2023.117335 37872010

[18] Jensen K, Nizamutdinov D, Guerrier M, Afroze S, Dostal D, Glaser S. General mechanisms of nicotine-induced fibrogenesis. FASEB J 2012;26:4778–87. 10.1096/fj.12-206458 22906950 PMC3509054

[19] Cooke JP, Bitterman H. Nicotine and angiogenesis: a new paradigm for tobacco-related diseases. Ann Med 2004;36:33–40. 10.1080/07853890310017576 15000345

[20] Barrington-Trimis JL, Leventhal AM. Adolescents’ use of “pod mod” E-cigarettes—urgent concerns. N Engl J Med 2018;379:1099–102. 10.1056/NEJMp1805758 30134127 PMC7489756

[21] Visseren FLJ, Mach F, Smulders YM, Carballo D, Koskinas KC, Back M, et al 2021 ESC guidelines on cardiovascular disease prevention in clinical practice. Eur Heart J 2021;42:3227–337. 10.1093/eurheartj/ehab484 34458905

[22] Dennison Himmelfarb CR, Benowitz NL, Blank MD, Bhatnagar A, Chase PJ, Davis EM, et al Impact of smokeless oral nicotine products on cardiovascular disease: implications for policy, prevention, and treatment: a policy statement from the American Heart Association. Circulation 2025;151:e1–21. 10.1161/cir.0000000000001293 39624904

[23] FDA. E-Cigarettes, Vapes, and other Electronic Nicotine Delivery Systems (ENDS). https://www.fda.gov/tobacco-products/products-ingredients-components/e-cigarettes-vapes-and-other-electronic-nicotine-deliverysystems-ends (24 October 2022, date last accessed).

[24] WHO. WHO report on the Global Tobacco Epidemic, 2025: Warning About the Dangers of Tobacco. Geneva: WHO, 2025.

[25] World Health Organization, American Heart Association, American College of Cardiology, and European Society of Cardiology. The tobacco endgame—addressing the cardiovascular harms of tobacco use: joint statement by the World Health Organization, American Heart Association, American College of Cardiology, and European Society of Cardiology. Glob Heart 2021;16:71. 10.5334/gh.107934900562

[26] Rodgman A, Perfetti T. The Chemical Components of Tobacco and Tobacco Smoke. Boca Raton: CRC Press, 2013.

[27] Hoffmann D, Hoffmann I. Letters to the Editor—tobacco smoke components. Beitr Tab Int 1998;18:49–52. 10.2478/cttr-2013-0668

[28] Goniewicz ML, Knysak J, Gawron M, Kosmider L, Sobczak A, Kurek J, et al Levels of selected carcinogens and toxicants in vapour from electronic cigarettes. Tob Control 2014;23:133–9. 10.1136/tobaccocontrol-2012-050859 23467656 PMC4154473

[29] Tayyarah R, Long GA. Comparison of select analytes in aerosol from e-cigarettes with smoke from conventional cigarettes and with ambient air. Regul Toxicol Pharmacol 2014;70:704–10. 10.1016/j.yrtph.2014.10.010 25444997

[30] Rezk-Hanna M, Rossman MJ, Ludwig K, Sakti P, Cheng CW, Brecht ML, et al Electronic hookah (waterpipe) vaping reduces vascular endothelial function: the role of nicotine. Am J Physiol Heart Circ Physiol 2024;326:H490–6. 10.1152/ajpheart.00710.2023 38133618 PMC11219048

[31] Fetterman JL, Keith RJ, Palmisano JN, McGlasson KL, Weisbrod RM, Majid S, et al Alterations in vascular function associated with the use of combustible and electronic cigarettes. J Am Heart Assoc 2020;9:e014570. 10.1161/JAHA.119.014570 32345096 PMC7428567

[32] Mohammadi L, Han DD, Xu F, Huang A, Derakhshandeh R, Rao P, et al Chronic e-cigarette use impairs endothelial function on the physiological and cellular levels. Arterioscler Thromb Vasc Biol 2022;42:1333–50. 10.1161/atvbaha.121.317749 36288290 PMC9625085

[33] George J, Hussain M, Vadiveloo T, Ireland S, Hopkinson P, Struthers AD, et al Cardiovascular effects of switching from tobacco cigarettes to electronic cigarettes. J Am Coll Cardiol 2019;74:3112–20. 10.1016/j.jacc.2019.09.067 31740017 PMC6928567

[34] Rezk-Hanna M, Gupta R, Nettle CO, Dobrin D, Cheng CW, Means A, et al Differential effects of electronic hookah vaping and traditional combustible hookah smoking on oxidation, inflammation, and arterial stiffness. Chest 2022;161:208–18. 10.1016/j.chest.2021.07.027 34298007 PMC8783031

[35] MacDonald A, Middlekauff HR. Electronic cigarettes and cardiovascular health: what do we know so far? Vasc Health Risk Manag 2019;15:159–74. 10.2147/VHRM.S175970 31417268 PMC6592370

[36] Mallock N, Boss L, Burk R, Danziger M, Welsch T, Hahn H, et al Levels of selected analytes in the emissions of “heat not burn” tobacco products that are relevant to assess human health risks. Arch Toxicol 2018;92:2145–9. 10.1007/s00204-018-2215-y 29730817 PMC6002459

[37] Li X, Luo Y, Jiang X, Zhang H, Zhu F, Hu S, et al Chemical analysis and simulated pyrolysis of tobacco heating system 2.2 compared to conventional cigarettes. Nicotine Tob Res 2019;21:111–8. 10.1093/ntr/nty005 29319815

[38] Loffredo L, Carnevale R, Battaglia S, Marti R, Pizzolo S, Bartimoccia S, et al Impact of chronic use of heat-not-burn cigarettes on oxidative stress, endothelial dysfunction and platelet activation: the SUR-VAPES Chronic Study. Thorax 2021;76:618–20. 10.1136/thoraxjnl-2020-215900 34157671

[39] Biondi-Zoccai G, Sciarretta S, Bullen C, Nocella C, Violi F, Loffredo L, et al Acute effects of heat-not-burn, electronic vaping, and traditional tobacco combustion cigarettes: the sapienza university of Rome-vascular assessment of proatherosclerotic effects of smoking (SUR—VAPES) 2 randomized trial. J Am Heart Assoc 2019;8:e010455. 10.1161/JAHA.118.010455 30879375 PMC6475061

[40] Rao P, Han DD, Tan K, Mohammadi L, Derakhshandeh R, Navabzadeh M, et al Comparable impairment of vascular endothelial function by a wide range of electronic nicotine delivery devices. Nicotine Tob Res 2022;24:1055–62. 10.1093/ntr/ntac019 35100430 PMC9199952

[41] Ikonomidis I, Vlastos D, Kostelli G, Kourea K, Katogiannis K, Tsoumani M, et al Differential effects of heat-not-burn and conventional cigarettes on coronary flow, myocardial and vascular function. Sci Rep 2021;11:11808. 10.1038/s41598-021-91245-9 34083663 PMC8175445

[42] Loffredo L, Carnevale R, Pannunzio A, Cinicola BL, Palumbo IM, Bartimoccia S, et al Impact of heat-not-burn cigarette passive smoking on children's oxidative stress, endothelial and platelet function. Environ Pollut 2024;345:123304. 10.1016/j.envpol.2024.123304 38295930

[43] Bhatnagar A, Maziak W, Eissenberg T, Ward KD, Thurston G, King BA, et al Water pipe (hookah) smoking and cardiovascular disease risk: a scientific statement from the American Heart Association. Circulation 2019;139:e917–36. 10.1161/CIR.0000000000000671 30845826 PMC6600812

[44] Shihadeh A, Schubert J, Klaiany J, El Sabban M, Luch A, Saliba NA. Toxicant content, physical properties and biological activity of waterpipe tobacco smoke and its tobacco-free alternatives. Tob Control 2015;24(Suppl 1):i22–30. 10.1136/tobaccocontrol-2014-051907 25666550 PMC4345918

[45] Selim GM, Elia RZ, El Bohey AS, El Meniawy KA. Effect of shisha vs. cigarette smoking on endothelial function by brachial artery duplex ultrasonography: an observational study. Anadolu Kardiyol Derg 2013;13:759–65. 10.5152/akd.2013.4499 24287354

[46] Rezk-Hanna M, Mosenifar Z, Benowitz NL, Rader F, Rashid M, Davoren K, et al High carbon monoxide levels from charcoal combustion mask acute endothelial dysfunction induced by hookah (waterpipe) smoking in young adults. Circulation 2019;139:2215–24. 10.1161/CIRCULATIONAHA.118.037375 30764644

[47] Chami HA, Diab M, Zaouk N, Arnaout S, Mitchell GF, Isma'eel H, et al Central and peripheral hemodynamics in young adults who use water pipes and the acute effects of water-pipe use. Chest 2023;164:1481–91. 10.1016/j.chest.2023.07.070 37541338

[48] Bentur L, Hellou E, Goldbart A, Pillar G, Monovich E, Salameh M, et al Laboratory and clinical acute effects of active and passive indoor group water-pipe (narghile) smoking. Chest 2014;145:803–9. 10.1378/chest.13-0960 24158379

[49] Ali M, Jawad M. Health effects of waterpipe tobacco use: getting the public health message just right. Tob Use Insights 2017;10:1179173X17696055. 10.1177/1179173(17696055 PMC5428225 28579844

[50] Tasdighi E, Yao Z, Jha KK, Dardari ZA, Osuji N, Rajan T, et al Cigar, pipe, and smokeless tobacco use and cardiovascular outcomes from cross cohort collaboration. JAMA Netw Open 2025;8:e2453987. 10.1001/jamanetworkopen.2024.53987 39804647 PMC11731180

[51] Rohani M, Agewall S. Oral snuff impairs endothelial function in healthy snuff users. J Intern Med 2004;255:379–83. 10.1046/j.1365-2796.2003.01279.x 14871462

[52] Arefalk G, Hambraeus K, Lind L, Michaëlsson K, Lindahl B, Sundström J. Discontinuation of smokeless tobacco and mortality risk after myocardial infarction. Circulation 2014;130:325–32. 10.1161/circulationaha.113.007252 24958793

[53] Byhamre ML, Araghi M, Alfredsson L, Bellocco R, Engström G, Eriksson M, et al Swedish snus use is associated with mortality: a pooled analysis of eight prospective studies. Int J Epidemiol 2021;49:2041–50. 10.1093/ije/dyaa197 33347584 PMC7825961

[54] Van't Hof JR, Wang W, Matsushita K, Heiss G, Folsom AR, Widome R, et al Association of smokeless tobacco use with incident peripheral artery disease: results from the Atherosclerotic Risk in Communities Study. Am J Prev Med 2023;64:728–33. 10.1016/j.amepre.2023.01.001 36682917 PMC10121742

[55] Mushtaq N, Sarwar Z, Kouplen K, Ahmed R, Beebe LA. Association of cardiovascular disease risk factors with exclusive smokeless tobacco use among us males: cross-sectional analysis of NHANES data 2003–2018. Am J Health Promot 2023;37:614–24. 10.1177/08901171221141980 36535915 PMC10434754

[56] Erhabor J, Boakye E, Obisesan O, Osei AD, Tasdighi E, Mirbolouk H, et al E-cigarette use among us adults in the 2021 behavioral risk factor surveillance system survey. JAMA Netw Open 2023;6:e2340859. 10.1001/jamanetworkopen.2023.40859 37921768 PMC10625038

[57] WHO. WHO releases first-ever clinical treatment guideline for tobacco cessation in adults. https://www.who.int/news/item/02-07-2024-who-releases-first-ever-clinical-treatment-guideline-for-tobacco-cessation-in-adults (22 May 2025, date last accessed). PMC11288497 39074891

[58] Birdsey J, Cornelius M, Jamal A, Park-Lee E, Cooper MR, Wang J, et al Tobacco product use among us middle and high school students—national youth tobacco survey, 2023. MMWR Morb Mortal Wkly Rep 2023;72:1173–82. 10.15585/mmwr.mm7244a1 37917558 PMC10629751

[59] Action on Smoking and Health. Use of vapes (e-cigarettes) among adults in Great Britain. https://ash.org.uk/uploads/Use-of-vapes-among-adults-in-Great-Britain-2024.pdf (4 October 2025, date last accessed).

[60] BMA. Taking our breath away: why we need stronger regulation of vapes. https://www.bma.org.uk/media/gibk2p2y/bma-vapereport-v5.pdf (20 July 2025, date last accessed).

[61] Prochaska JJ, Vogel EA, Benowitz N. Nicotine delivery and cigarette equivalents from vaping a JUULpod. Tob Control 2022;31:e88–93. 10.1136/tobaccocontrol-2020-056367 33762429 PMC8460696

[62] Jerzyński T, Stimson GV, Shapiro H, Król G. Estimation of the global number of e-cigarette users in 2020. Harm Reduct J 2021;18:109. 10.1186/s12954-021-00556-7 34688284 PMC8541798

[63] Leventhal AM, Dai H, Barrington-Trimis JL, Tackett AP, Pedersen ER, Tran DD. Disposable e-cigarette use prevalence, correlates, and associations with previous tobacco product use in young adults. Nicotine Tob Res 2022;24:372–9. 10.1093/ntr/ntab165 34379787 PMC8842396

[64] Hammond D, Reid JL, Cole AG, Leatherdale ST. Electronic cigarette use and smoking initiation among youth: a longitudinal cohort study. CMAJ 2017;189:E1328–36. 10.1503/cmaj.161002 29084759 PMC5662449

[65] Chaffee BW, Couch ET, Wilkinson ML, Donaldson CD, Cheng NF, Ameli N, et al Flavors increase adolescents’ willingness to try nicotine and cannabis vape products. Drug Alcohol Depend 2023;246:109834. 10.1016/j.drugalcdep.2023.109834 36963159 PMC10121941

[66] Kang D, Choi KH, Kim H, Park H, Heo J, Park TK, et al Prognosis after switching to electronic cigarettes following percutaneous coronary intervention: a Korean nationwide study. Eur Heart J 2024;46:84–95. 10.1093/eurheartj/ehae705 39429032

[67] Lindson N, Theodoulou A, Ordóñez-Mena JM, Fanshawe TR, Sutton AJ, Livingstone-Banks J, et al Pharmacological and electronic cigarette interventions for smoking cessation in adults: component network meta-analyses. Cochrane Database Syst Rev 2023;9:CD015226. 10.1002/14651858.CD015226.pub2 37696529 PMC10495240

[68] Hajek P, Phillips-Waller A, Przulj D, Pesola F, Myers Smith K, Bisal N, et al A randomized trial of e-cigarettes versus nicotine-replacement therapy. N Engl J Med 2019;380:629–37. 10.1056/NEJMoa1808779 30699054

[69] Munzel T, Daiber A, Prochaska J. How quitting, switching to e-cigarettes, or sticking to smoking shapes cardiovascular outcomes after percutaneous coronary intervention. Eur Heart J 2025;46:96–8. 10.1093/eurheartj/ehae756 39523565

[70] Office for Health Improvement and Disparities. Nicotine vaping in England: 2022 evidence update main findings. https://www.gov.uk/government/publications/nicotine-vaping-in-england-2022-evidence-update/nicotine-vaping-in-england-2022-evidence-update-main-findings (23 May 2025, date last accessed).

[71] WHO. Tobacco use and exposure to second-hand smoke linked to more than 20% of deaths from coronary heart disease. https://www.who.int/europe/news/item/29-09-2020-tobacco-use-and-exposure-to-second-hand-smoke-linked-to-more-than-20-of-deaths-from-coronary-heart-disease (11 June 2025, date last accessed).

[72] Celermajer DS, Adams MR, Clarkson P, Robinson J, McCredie R, Donald A, et al Passive smoking and impaired endothelium-dependent arterial dilatation in healthy young adults. N Engl J Med 1996;334:150–4. 10.1056/NEJM199601183340303 8531969

[73] Bostrom CE, Gerde P, Hanberg A, Jernstrom B, Johansson C, Kyrklund T, et al Cancer risk assessment, indicators, and guidelines for polycyclic aromatic hydrocarbons in the ambient air. Environ Health Perspect 2002;110(Suppl 3):451–88. 10.1289/ehp.110-1241197 12060843 PMC1241197

[74] CDC. Health Effects of Vaping. Atlanta, Georgia: CDC, 2025.

[75] El-Mahdy MA, Ewees MG, Eid MS, Mahgoup EM, Khaleel SA, Zweier JL. Electronic cigarette exposure causes vascular endothelial dysfunction due to NADPH oxidase activation and eNOS uncoupling. Am J Physiol Heart Circ Physiol 2022;322:H549–67. 10.1152/ajpheart.00460.2021 35089811 PMC8917923

[76] ESC. European Society of Cardiology: Vaping must be included in the next EU smoking ban. https://www.escardio.org/The-ESC/Press-Office/Press-releases/vaping-must-be-included-in-the-eu-smoking-ban (15 October 2025, date last accessed).

[77] WHO. Electronic Cigarettes (E-Cigarrettes). Geneva: WHO, 2024.

[78] Shihadeh A, Azar S, Antonios C, Haddad A. Towards a topographical model of narghile water-pipe café smoking: a pilot study in a high socioeconomic status neighborhood of Beirut, Lebanon. Pharmacol Biochem Behav 2004;79:75–82. 10.1016/j.pbb.2004.06.005 15388286

[79] Wang L, Liu X, Chen L, Liu D, Yu T, Bai R, et al Harmful chemicals of heat not burn product and its induced oxidative stress of macrophages at air-liquid interface: comparison with ultra-light cigarette. Toxicol Lett 2020;331:200–7. 10.1016/j.toxlet.2020.06.017 32569802

[80] Celermajer DS, Sorensen KE, Georgakopoulos D, Bull C, Thomas O, Robinson J, et al Cigarette smoking is associated with dose-related and potentially reversible impairment of endothelium-dependent dilation in healthy young adults. Circulation 1993;88:2149–55. 10.1161/01.CIR.88.5.2149 8222109

[81] Heitzer T, Just H, Münzel T. Antioxidant vitamin C improves endothelial dysfunction in chronic smokers. Circulation 1996;94:6–9. 10.1161/01.cir.94.1.6 8964118

[82] Skaug EA, Nes B, Aspenes ST, Ellingsen O. Non-smoking tobacco affects endothelial function in healthy men in one of the largest health studies ever performed; the nord-trondelag health study in Norway; HUNT3. PLoS One 2016;11:e0160205. 10.1371/journal.pone.0160205 27490361 PMC4974005

[83] Heitzer T, Brockhoff C, Mayer B, Warnholtz A, Mollnau H, Henne S, et al Tetrahydrobiopterin improves endothelium-dependent vasodilation in chronic smokers: evidence for a dysfunctional nitric oxide synthase. Circ Res 2000;86:E36–41. 10.1161/01.res.86.2.e36 10666424

[84] Lerman A, Zeiher AM. Endothelial function: cardiac events. Circulation 2005;111:363–8. 10.1161/01.CIR.0000153339.27064.14 15668353

[85] Olfert IM, DeVallance E, Hoskinson H, Branyan KW, Clayton S, Pitzer CR, et al Chronic exposure to electronic cigarettes results in impaired cardiovascular function in mice. J Appl Physiol (1985) 2018;124:573–82. 10.1152/japplphysiol.00713.2017 29097631 PMC5899271

[86] Rao P, Liu J, Springer ML. JUUL and combusted cigarettes comparably impair endothelial function. Tob Regul Sci 2020;6:30–7. 10.18001/TRS.6.1.4 31930162 PMC6953758

[87] Teo KK, Ounpuu S, Hawken S, Pandey MR, Valentin V, Hunt D, et al Tobacco use and risk of myocardial infarction in 52 countries in the INTERHEART study: a case-control study. Lancet 2006;368:647–58. 10.1016/S0140-6736(06)69249-0 16920470

[88] Mons U, Muezzinler A, Gellert C, Schottker B, Abnet CC, Bobak M, et al Impact of smoking and smoking cessation on cardiovascular events and mortality among older adults: meta-analysis of individual participant data from prospective cohort studies of the CHANCES consortium. BMJ 2015;350:h1551. 10.1136/bmj.h1551 25896935 PMC4413837

[89] Ding N, Sang Y, Chen J, Ballew SH, Kalbaugh CA, Salameh MJ, et al Cigarette smoking, smoking cessation, and long-term risk of 3 major atherosclerotic diseases. J Am Coll Cardiol 2019;74:498–507. 10.1016/j.jacc.2019.05.049 31345423 PMC6662625

[90] Alzahrani T, Pena I, Temesgen N, Glantz SA. Association between electronic cigarette use and myocardial infarction. Am J Prev Med 2018;55:455–61. 10.1016/j.amepre.2018.05.004 30166079 PMC6208321

[91] Farsalinos KE, Polosa R, Cibella F, Niaura R. Is e-cigarette use associated with coronary heart disease and myocardial infarction? Insights from the 2016 and 2017 National Health Interview Surveys. Ther Adv Chronic Dis 2019;10:2040622319877741. 10.1177/2040622319877741 PMC6767743 31632622

[92] Waziry R, Jawad M, Ballout RA, Al Akel M, Akl EA. The effects of waterpipe tobacco smoking on health outcomes: an updated systematic review and meta-analysis. Int J Epidemiol 2017;46:32–43. 10.1093/ije/dyw021 27075769

[93] Sibai AM, Tohme RA, Almedawar MM, Itani T, Yassine SI, Nohra EA, et al Lifetime cumulative exposure to waterpipe smoking is associated with coronary artery disease. Atherosclerosis 2014;234:454–60. 10.1016/j.atherosclerosis.2014.03.036 24814409

[94] Kinnunen JM, Rimpelä AH, Lindfors PL, Clancy L, Alves J, Hoffmann L, et al Electronic cigarette use among 14- to 17-year-olds in Europe. Eur J Public Health 2021;31:402–8. 10.1093/eurpub/ckaa145 33079986 PMC8071596

[95] Kavousi M, Pisinger C, Barthelemy JC, De Smedt D, Koskinas K, Marques-Vidal P, et al Electronic cigarettes and health with special focus on cardiovascular effects: position paper of the European Association of Preventive Cardiology (EAPC). Eur J Prev Cardiol 2021;28:1552–66. 10.1177/2047487320941993 32726563

[96] Smith MJ, Buckton C, Patterson C, Hilton S. User-generated content and influencer marketing involving e-cigarettes on social media: a scoping review and content analysis of YouTube and Instagram. BMC Public Health 2023;23:530. 10.1186/s12889-023-15389-1 36941553 PMC10029293

[97] Mallock-Ohnesorg N, Rabenstein A, Stoll Y, Gertzen M, Rieder B, Malke S, et al Small pouches, but high nicotine doses-nicotine delivery and acute effects after use of tobacco-free nicotine pouches. Front Pharmacol 2024;15:1392027. 10.3389/fphar.2024.1392027 38841367 PMC11150668

[98] WHO. WHO clinical treatment guideline for tobacco cessation in adults. https://www.who.int/publications/i/item/9789240096431 (11 June 2025, date last accessed).

[99] Celermajer DS, Sorensen KE, Gooch VM, Spiegelhalter DJ, Miller OI, Sullivan ID, et al Non-invasive detection of endothelial dysfunction in children and adults at risk of atherosclerosis. Lancet 1992;340:1111–5. 10.1016/0140-6736(92)93147-f 1359209

[100] EEA. Europe's environment 2025. https://www.eea.europa.eu/en/europe-environment-2025 (9 October 2025, date last accessed).

[101] Rezk-Hanna M, Doering L, Robbins W, Sarna L, Elashoff RM, Victor RG. Acute effect of hookah smoking on arterial stiffness and wave reflections in adults aged 18 to 34 years of age. Am J Cardiol 2018;122:905–9. 10.1016/j.amjcard.2018.05.033 30057235

[102] Sheikh ZD, Branston JR, Olefir L, Welding K. Examining cigarette, heated tobacco and e-cigarette market pricing and tax pass-through in Ukraine during the 2019–2022 tax reforms. Tob Control 2025:tc-2025-059290. 10.1136/tc-2025-059290 40360289

[103] Ruth Canty. The Ploom case: defying German law. https://blogs.bmj.com/tc/2025/03/21/the-ploomcase-defying-german-law/ (posted March 21, 2025).

